# Human iPSC-derived MSCs (iMSCs) from aged individuals acquire a rejuvenation signature

**DOI:** 10.1186/s13287-019-1209-x

**Published:** 2019-03-18

**Authors:** Lucas-Sebastian Spitzhorn, Matthias Megges, Wasco Wruck, Md Shaifur Rahman, Jörg Otte, Özer Degistirici, Roland Meisel, Rüdiger Volker Sorg, Richard O. C. Oreffo, James Adjaye

**Affiliations:** 1 Institute for Stem Cell Research and Regenerative Medicine, Medical Faculty, Heinrich Heine University, Düsseldorf, Moorenstr. 5, 40225 Düsseldorf, Germany; 2 Division of Paediatric Stem Cell Therapy, Clinic for Pediatric Oncology, Hematology and Clinical Immunology, Medical Faculty, Heinrich Heine University, Düsseldorf, Moorenstr. 5, 40225 Düsseldorf, Germany; 3 Institute for Transplantation Diagnostics and Cell Therapeutics, Heinrich Heine University Hospital, Moorenstr, 5, 40225 Düsseldorf, Germany; 4 Bone and Joint Research Group, Centre for Human Development, Stem Cells and Regeneration, Institute of Developmental Sciences, University of Southampton, Southampton, SO16 6YD UK

**Keywords:** Aged MSC, Fetal MSCs, iPSCs, iMSCs, Transcriptome, Secretome, Rejuvenation, Aging

## Abstract

**Background:**

Primary mesenchymal stem cells (MSCs) are fraught with aging-related shortfalls. Human-induced pluripotent stem cell (iPSC)-derived MSCs (iMSCs) have been shown to be a useful clinically relevant source of MSCs that circumvent these aging-associated drawbacks. To date, the extent of the retention of aging-hallmarks in iMSCs differentiated from iPSCs derived from elderly donors remains unclear.

**Methods:**

Fetal femur-derived MSCs (fMSCs) and adult bone marrow MSCs (aMSCs) were isolated, corresponding iPSCs were generated, and iMSCs were differentiated from fMSC-iPSCs, from aMSC-iPSCs, and from human embryonic stem cells (ESCs) H1. In addition, typical MSC characterization such as cell surface marker expression, differentiation capacity, secretome profile, and trancriptome analysis were conducted for the three distinct iMSC preparations—fMSC-iMSCs, aMSC-iMSCs, and ESC-iMSCs. To verify these results, previously published data sets were used, and also, additional aMSCs and iMSCs were analyzed.

**Results:**

fMSCs and aMSCs both express the typical MSC cell surface markers and can be differentiated into osteogenic, adipogenic, and chondrogenic lineages in vitro*.* However, the transcriptome analysis revealed overlapping and distinct gene expression patterns and showed that fMSCs express more genes in common with ESCs than with aMSCs. fMSC-iMSCs, aMSC-iMSCs, and ESC-iMSCs met the criteria set out for MSCs. Dendrogram analyses confirmed that the transcriptomes of all iMSCs clustered together with the parental MSCs and separated from the MSC-iPSCs and ESCs. iMSCs irrespective of donor age and cell type acquired a rejuvenation-associated gene signature, specifically, the expression of *INHBE*, *DNMT3B*, *POU5F1P1*, *CDKN1C*, and *GCNT2* which are also expressed in pluripotent stem cells (iPSCs and ESC) but not in the parental aMSCs. iMSCs expressed more genes in common with fMSCs than with aMSCs. Independent real-time PCR comparing aMSCs, fMSCs, and iMSCs confirmed the differential expression of the rejuvenation (*COX7A*, *EZA2*, and *TMEM119*) and aging (*CXADR* and *IGSF3*) signatures. Importantly, in terms of regenerative medicine, iMSCs acquired a secretome (e.g., angiogenin, DKK-1, IL-8, PDGF-AA, osteopontin, SERPINE1, and VEGF) similar to that of fMSCs and aMSCs, thus highlighting their ability to act via paracrine signaling.

**Conclusions:**

iMSCs irrespective of donor age and cell source acquire a rejuvenation gene signature. The iMSC concept could allow circumventing the drawbacks associated with the use of adult MSCs und thus provide a promising tool for use in various clinical settings in the future.

**Electronic supplementary material:**

The online version of this article (10.1186/s13287-019-1209-x) contains supplementary material, which is available to authorized users.

## Background

Primary human bone marrow-derived stem cells (MSCs) contain a sub-population of multipotent stem cells which retain osteogenic, chondrogenic, and adipogenic differentiation potential [1, 2]. Apart from the adult sources, these multipotent MSCs have been isolated from fetal femur [3]. Due to highly proliferative, immune-modulatory properties, and paracrine orchestration, MSCs offer significant therapeutic potential for an increasing aging demographic [4].

Although the bone marrow can be collected routinely to isolate MSCs, there are several drawbacks associated with the use of MSCs from aged individuals. Aging involves enhanced cellular senescence, instability of the genome, accumulation of DNA damage, changes in DNA repair pathways, oxidative stress, metabolic instability, and activated immune response [5–8]. In line with this, the expansion possibilities and application potential of primary MSCs are limited, in part, by changes in the differentiation/response potential and function of MSCs isolated from aged donors [9–11]. However, to date, it remains unclear whether there are any age-related differences in transcriptome and secretome signatures between human fetal MSCs and MSCs from elderly donors.

Recent studies have shown that the shortfalls associated with primary MSCs can be circumvented by reprogramming them to induced pluripotent stem cells (iPSCs) [12–14]. iPSCs have the potential to self-renew, bypass senescence and are similar to human embryonic stem cells (ESCs). However, the parental somatic aging signature and secretome properties and subsequent reflection in iPSC derivatives are unknown [15–17]. An iPSC-derived cell type that is of prime interest for circumventing shortfalls associated with primary MSCs are MSCs differentiated from iPSCs and ESCs (iMSCs). The similarity of iMSCs to primary MSCs and their regenerative potential in vivo has already been demonstrated [18, 19]. Moreover, the reflection of donor age in iMSCs was shown to be reverted into a younger state and at the same time reflected in iMSCs from patients with early onset aging syndromes [13, 20]. Although the paracrine effects of iMSCs have been indicated [21], relatively little is known about the potential to rejuvenate the paracrine features of MSCs from elderly patients via iMSC generation.

In view of this, there is a dire need to clarify in more detail whether age-related features inherent to primary MSCs isolated from elderly patients are retained in the corresponding iMSCs at the transcriptional, secretome, and functional level. In this study, we report the age-associated differences between fetal MSC (fMSC) populations and MSCs isolated from elderly donors with respect to their transcriptomes. We successfully reprogrammed fMSCs (55 days post conception) and adult MSC (aMSC; 60–74 years) to iPSCs and, subsequently, generated the corresponding iMSCs. In addition, iMSCs were also derived from ESCs. The iMSCs were similar although not identical to primary MSCs. We unraveled a putative rejuvenation and aging gene expression signature. We show that iMSCs irrespective of donor age and cell type re-acquired a similar secretome to that of their parental MSCs, thus re-enforcing their capabilities of context-dependent paracrine signaling relevant for tissue regeneration.

## Methods

### MSC preparations used in this study

Fetal femur-derived MSCs were obtained at 55 days post-conception as previously described [3] following informed, written patient consent. Approval was obtained by the Southampton and South West Hampshire Local Research Ethics Committee (LREC 296100). Mesenchymal stem cells, used for generation of iPSCs and iMSCs, were isolated from the bone marrow of a 74-year-old female donor as described before [22] after written informed consent. The corresponding protocol was approved by the research ethics board of the Charite-Universitätsmedizin, Berlin (IRB approval EA2/126/07). Aged MSCs (60 years, 62 years, and 70 years) were isolated as previously described [23]. Isolation of mesenchymal stem cells from 60 to 70-year-old individuals was approved under the Southampton and South West Hampshire Local Research Ethics Committee (LREC 194/99). Three primary fetal MSC preparations, fMSC1, fMSC2, and fMSC3, derived from different donors, were compared to MSCs isolated from elderly donors between 60 and 74 years of age; aMSC1, aMSC2, aMSC3, and aMSC4 (Additional file 1: Table S1). For meta-analyses, we included published transcriptome datasets of adult human MSCs which are referred to as MSC1, MSC2, and MSC3 [24] and adult MSCs from donors aged 29, 48, 60, and 76 years are referred to as MSC4, MSC5, MSC6, and MSC7 [25]. For the purpose of comparing the secretomes of iMSCs, fMSCs, and aMSCs, three additional MSC preparations from donors aged 62, 64, and 69 years were used, which had been generated and characterized (data not shown) at the Institute for Transplantation Diagnostics and Cell Therapeutics at Heinrich Heine University Hospital, Düsseldorf with patient consent and approval of the Ethics commission of the medical faculty Heinrich Heine University (Study number: 5013).

### Cell culture

The culture of MSCs and iMSCs was carried out in αMEM, nucleosides, GlutaMAX with addition of 10% fetal bovine serum (Biochrom AG, Germany), penicillin/streptomycin, and nonessential amino acids (all from Life Technologies, California, USA). MSCs and iMSCs were expanded with a seeding density of 1000 cells per cm^2^. iMSCs were cultured in the same conditions starting from passage four [22].

Pluripotent stem cells (iPSCs and ESCs H1 and H9 (#WA01 and #WA09, respectively)) were cultured in unconditioned medium. The medium contained KO-DMEM, supplementation of 20% serum replacement, sodium pyruvate, nonessential amino acids, l-glutamine, penicillin/streptomycin, and 0 .1mM β-mercaptoethanol (all from Life Technologies). Supplementation with basic fibroblast growth factor (bFGF) (Preprotech, USA) to a final concentration of 8 ng/ml was carried out before media change every day. Passaging of pluripotent stem cells was carried out with a splitting ratio of 1:3 to 1:10. Passaging was conducted manually using a syringe needle and a pipette under a binocular microscope or using a cell scraper and PBS (−). Mitomycin-C inactivated mouse embryonic fibroblasts were used as feeder cells seeded on cell culture dishes coated with Matrigel (BD) to culture iPSCs and ESCs. MSC culture was carried out at 37 °C and 5% CO_2_ in a humidified atmosphere. Pluripotent stem cell culture was carried out under the same condition with additional hypoxic conditions in 5% O_2_ [26].

### In vitro differentiation of parental MSCs and iMSCs

Adipocyte differentiation was carried out using the StemPro Adipogenesis Differentiation Kit (Life technologies, USA). The MSCs were seeded at an initial density of 1 × 10^4^ cells per cm^2^ and induced to the adipogenic fate with differentiation medium and cultured for 21 days. Lipid filled vacuoles were visualized with Oil red O after adipogenic induction. Osteoblast differentiation was performed with the StemPro Osteogenesis Differentiation Kit (Life Technologies). Calcified matrix was visualized with Alizarin Red after osteogenic induction. The MSCs were seeded at a density of 5 × 10^3^ cells per cm^2^ in osteogenic induction media and cultured for 21 days. Chondrocyte differentiation was carried out using StemPro Chondrogenesis Differentiation Kit (Life Technologies). Acidic mucosubstances were visualized by Alcian Blue staining after chondrogenic induction.

### Derivation of iPSCs from MSCs

fMSC-iPSC1, fMSC-iPSC2, and aMSC-iPSC1 were generated as previously described [22, 26]. Retroviral pluripotency induction in fetal femur-derived MSCs was carried out using pMX vector-based expression of *OCT4*, *SOX2*, *KLF4*, and *c-MYC*. Retrovirus generation was carried out in Phoenix cells using FuGENE HD Transfection Reagent (PROMEGA). Two hundred thousand MSCs were transduced. After transduction, MSCs were seeded onto Matrigel-coated cell culture plates with feeder cells (inactivated MEFs) at a density of 4000 cells per cm^2^ for pluripotency induction. To reprogram them, the transduced MSCs were culture in N2B27-based medium with additions of 20% serum replacement, sodium pyruvate, nonessential amino acids, l-glutamine, penicillin/streptomycin, and 0 .1mM β-mercaptoethanol (all from Life Technologies, USA) and bFGF (Preprotech, USA). After 14 days, the media was switched to mTeSR1 (Stem Cell Technologies, USA) as previously described [27]. The cells were cultured until ESC-like colonies became visible. The colonies were isolated manually and expanded for characterization. The resulting iPSCs were termed fMSC-iPSC3.

So termed, aMSC-iPSC2, were generated by using episomal plasmid-based reprogramming using the previously described combination of episomal plasmids 7F-2 [27]. The plasmids were delivered to aMSCs (62 years) by nucleofection using the Human MSC (Mesenchymal Stem Cell) Nucleofector Kit (Lonza, VPE-1001) and the Amaxa Nucleofector II (Lonza) following the manufacturer’s instructions. aMSCs were cultured until passage two and the combination of 3 μg of pEP4 EO2S EN2K, 3 .2 μg of pEP4 EO2S ET2K, and 2 .4 μg of pCEP4-M2L was mixed with the 1 × 10^6^ MSCs and nucleofected using the program U-23. Nucleofected aMSCs were expanded in MSC medium for 6 days and replated with a density of 6 × 10^4^ cells per well of a six-well plate onto Matrigel and feeder cell-coated culture vessels. Further culture was carried out in N2B27-based medium as already described. Fifty micrograms per milliliter of l-ascorbic acid (Sigma-Aldrich) was added to the medium [28] with a media change every other day. After 14 days, the media was switched to mTeSR1 (Stem Cell Technologies) as previously described [27]. Further culture was carried out until ESC-like colonies could be isolated. ESC-like cell colonies were isolated manually with a syringe needle and pipette under a stereo microscope.

The isolated colonies were seeded onto freshly prepared feeder cell-coated plates as described previously [29, 30]. The characterization of the iPSC clones was initiated after six passages. The isolated iPSCs colonies were characterized as previously described [22]. The pluripotency of iPSCs generated from MSCs was tested in a similar fashion as previously described for the tool PluriTest (http://www.pluritest.org) [31] by cluster analysis within the R statistical programming environment [32] using function *hclust* to show similarity with embryonal stem cells within a dendrogram.

### Embryoid body-based in vitro differentiation

iPSCs were seeded into low attachment culture dishes (Corning) and cultured in DMEM with additional 10% fetal bovine serum (Biochrom AG), sodium pyruvate, l-glutamine, nonessential amino acids, and penicillin/streptomycin (all from Life Technologies) without bFGF for the generation of embryoid bodies (EBs). EBs were transferred onto gelatin-coated culture dishes after 10 days and cultured further for 10 days using the same conditions. Next, the cells were fixed in 4% paraformaldehyde (PFA) and stained using immunofluorescence-based detection of germ layer-specific markers.

### Generation of iMSCs

iMSCs were generated from iPSCs and ESC line H1 as previously described [18]. In brief, iPSCs and ESCs were cultured without feeder cells on Matrigel. When confluency was reached, the medium was switched to unconditioned medium without bFGF supplementation or αMEM and with addition of 10 μM SB-431542 (Sigma-Aldrich) with a media change every day for 10 days. Next, the cells were trypsinized and seeded at a density of 4 × 10^4^ cells per cm^2^ onto uncoated culture dishes in MSC expansion medium. Subsequently, the cells were passaged and reseeded at a density of 2 × 10^4^ cells per cm^2^ under the same culture conditions. Finally, the cells were passaged and seeded at a density of 1 × 10^4^ cells per cm^2^. The seeding density was maintained in every further passage.

### Flow cytometry

The surface marker expression of MSCs and iMSCs was analyzed using MSC Phenotyping Kit (Miltenyi). The cells were trypsinized, washed with PBS and stained with labeled antibodies as well as analyzed according to the manufacturer’s instructions. For the analysis of the stained cells, fluorescence-activated cell sorting (FACS) calibur (BD) flow cytometer was used, the program CellQuestPro for data acquisition, and the softwares Cyflogic (http://www.cyflogic.com) and Microsoft Excel for data analysis.

### Quantitative real-time polymerase chain reaction

The Power SYBR Green Master Mix (Life technologies) was used for quantitative real-time PCR analysis. Three hundred eighty-four-well format plates were used, and the reaction mixture had final volume of 10 μl as recommended in the manufacturer’s protocol. An amount of 10 ng of cDNA was used for each reaction. The experiments were done in technical replicates. The ViiA7 (Life technologies) system was used to run the PCR with these conditions: 95°C for 10 min; 35 cycles of 95 °C, 60 °C, and 72 °C with 30 s each step. Melting curves were generated after all cycles were completed. The ^(−delta delta Ct) method was used to calculate relative gene expression levels using the CT mean values as an input. Normalization was done based on the housekeeping gene RPL37A. Table S2 shows primer sequences.

### Immunofluorescence staining

Immunofluorescence staining was used to detect pluripotency markers in iPSCs and to detect expression of germ layer-specific marker in cells differentiated from iPSCs in an embryonic body-based in vitro pluripotency test. The cells were fixed at room temperature with 4% PFA for 20 min. Subsequently, the cells were washed three times in PBS and incubated in 1% Triton X-100 in PBS for 10 min at room temperature. Next, the cells were incubated in blocking solution: 10% FCS (Vector) and 0.1% Triton X-100 (Sigma-Aldrich) in PBS for 1 h. Then, the cells were incubated with the primary antibody at 4 °C in blocking solution overnight followed by three washes with PBS. Next, the cells were incubated with the secondary antibody at room temperature for 1 h followed by three more washes with PBS and a final incubation with DAPI (200 ng/ml, Invitrogen) in PBS for 20 min at room temperature. This was followed by image acquisition using a using the confocal microscope LSM510 (Carl Zeiss). A list of the used antibodies is provided in the supplement (Additional file 1: Table S3).

### Gene expression analysis

The DNA+RNA+Protein Extraction Kit (Roboklon) was used to extract total RNA. The linear amplification kit (Ambion) was used to produce biotin-labeled cRNA form 500 ng of total RNA per sample. The samples were further processed using the Illumina BeadStation 500 platform (Illumina) following the manufacturer’s protocol for hybridization and Cy3-streptavidin staining. HumanHT-12 v3.0 Gene Expression Bead Chips (Illumina) were used to hybridize the samples. Bead-level data was summarized to bead-summary data using the Gene Expression Module of the software GenomeStudio (Illumina) without normalization and background correction. Bead-summary data was imported into the R/Bioconductor [33] environment where it was background-corrected and normalized with quantile normalization from the package *lumi* [34]. The R_builtin function *cor* was used to compute the Pearson correlation values between the transcriptomes detected by microarray. Significant gene expression was calculated by determining the detection *p* value based on the difference to negative control beads. A gene with a detection *p* value ≤ 0.05 was considered to be expressed. Venn diagrams and heatmaps were generated employing the R/Bioconductor packages VennDiagram [35] and gplots [36]. Lists of human gene sets annotated to the Gene Ontology (GO)-terms cell cycle, senescence, response to oxidative stress, DNA damage repair, and aging were generated using AmiGO 2 version 2.3.1 (http://amigo2.berkeleybop.org/amigo) [37] and used to extract GO term-specific gene expression data sets which were analyzed by hierarchical clustering analysis. The data set of the gene expression analysis will be accessible at the Gene Expression Omnibus (GEO) repository under the accession number GSE97311 (www.ncbi.nlm.nih.gov/geo/query/acc.cgi?acc=GSE97311).

### Determination of differential expression

The linear models for microarrays from the R/Bioconductor *limma* package [38] were used to compute the differential *p* value to determine the significance of the difference between gene expression values. The computed differential *p* value was adjusted in R/Bioconductor with the *qvalue* false discovery rate (FDR) correction algorithm [39]. Genes with a FDR-corrected differential *p* value of ≤ 0.05 were considered significantly different. The up- or downregulation of these genes was calculated by determining the ratio of the average signals. A ratio higher than 1.33 was considered as upregulated, and a ratio lower than 0.75 was considered as downregulated.

### Determination of the aged and rejuvenation gene signatures

Two gene signatures were determined which are characterizing the aging and rejuvenation processes. The gene signatures were identified based on combinations of gene expression of MSCs in differing age-related stages. The aged gene signature was defined by gene expression in the MSCs but not in the iMSCs and iPSCs, given by the combination of detection *p* values: *p*__MSC_ < 0.001Λ *p*__iMSC_ > 0.1Λ *p*__iPSC_ > 0.1. The rejuvenation gene signature was defined by gene expression in the iMSCs and iPSCs but not in the MSCs, given by the combination of detection *p* values: *p*__MSC_ > 0.1Λ *p*__iMSC_ < 0.001Λ *p*__iPSC_ < 0.001.

### Functional annotation of gene sets

Database for Annotation, Visualization and Integrated Discovery (DAVID) Bioinformatics resources 6.7 (http://david.abcc.ncifcrf.gov) [40] was used for functional annotation analysis of gene sets. Lists of official gene symbols or Illumina IDs were used as input against human background. The default settings of DAVID Bioinformatics resources 6.7 were used. The option Kyoto Encyclopedia of Genes and Genomes (KEGG) terms was used for the annotation to pathways. The option GO_BP_Direct or GO_BP_Fat was used to for annotation to gene ontology terms of biological processes.

### Protein association network

Based on the aged and rejuvenation gene signatures, two protein interaction networks were constructed. The interactions are based upon the Biogrid database version 3.4.161 [41] filtered for the taxonomy id 9606 (*Homo sapiens*). From the Biogrid dataset, all protein interactions containing at least one protein coded by the above mentioned aged and rejuvenation signatures were extracted separately for each signature. Both resulting networks were reduced by adding only the *n* = 30 interacting proteins with the most interactions to proteins coded by genes from the original sets. The R package *network* [42] was employed to visualize these interactions marking proteins from the original sets in green. Communities of related proteins within the networks were detected via an in-betweenness clustering analysis with the method *cluster_edge_betweenness* from the R package *igraph* [43].

### Secretome analysis

The cell culture supernatants of three distinct fMSCs, three independent iMSCs, and three distinct aMSCs were collected, and 1 .5 ml each was used for subsequent analysis. The Proteome ProfilerTM Array Human (XL) Cytokine Array kit (R&D Systems, catalog number ARY022) was carried out according to the user’s manual. Two reference spots showing successful performed analysis were located in three positions on the cytokine membrane (in upper left, lower left, and the lower right- corner). Horseradish peroxidase substrate and luminol enhancer solution (GE healthcare UK limited) were used to visualize protein distribution and amount on the membranes. The pictures were taken with Fusion-FX microscope (Fischer Biotec). The pixel density of the spots was measured using ImageJ, the background intensity was subtracted, and the values were finally calculated as percentage of the reference spots intensity. Values above 5% were classified as secreted. Cytokines with values above 20% of the reference were considered abundantly expressed.

### Statistical analysis

The comparison of two groups was carried out using a two-tailed unpaired Student’s *t* test. Significant difference was defined with *p* values ≤ 0.05. For microarray data analysis, a gene with an expression *p* value ≤ 0.01 was considered significantly expressed. A gene with a differential *p* value ≤ 0.01 was considered significantly different in terms of expression. Functional annotation was considered significant with a *p* value of ≤ 0.05.

## Results

### Mesenchymal stem cells of fetal and aged background differ in transcriptome level

**Fig. 1:**
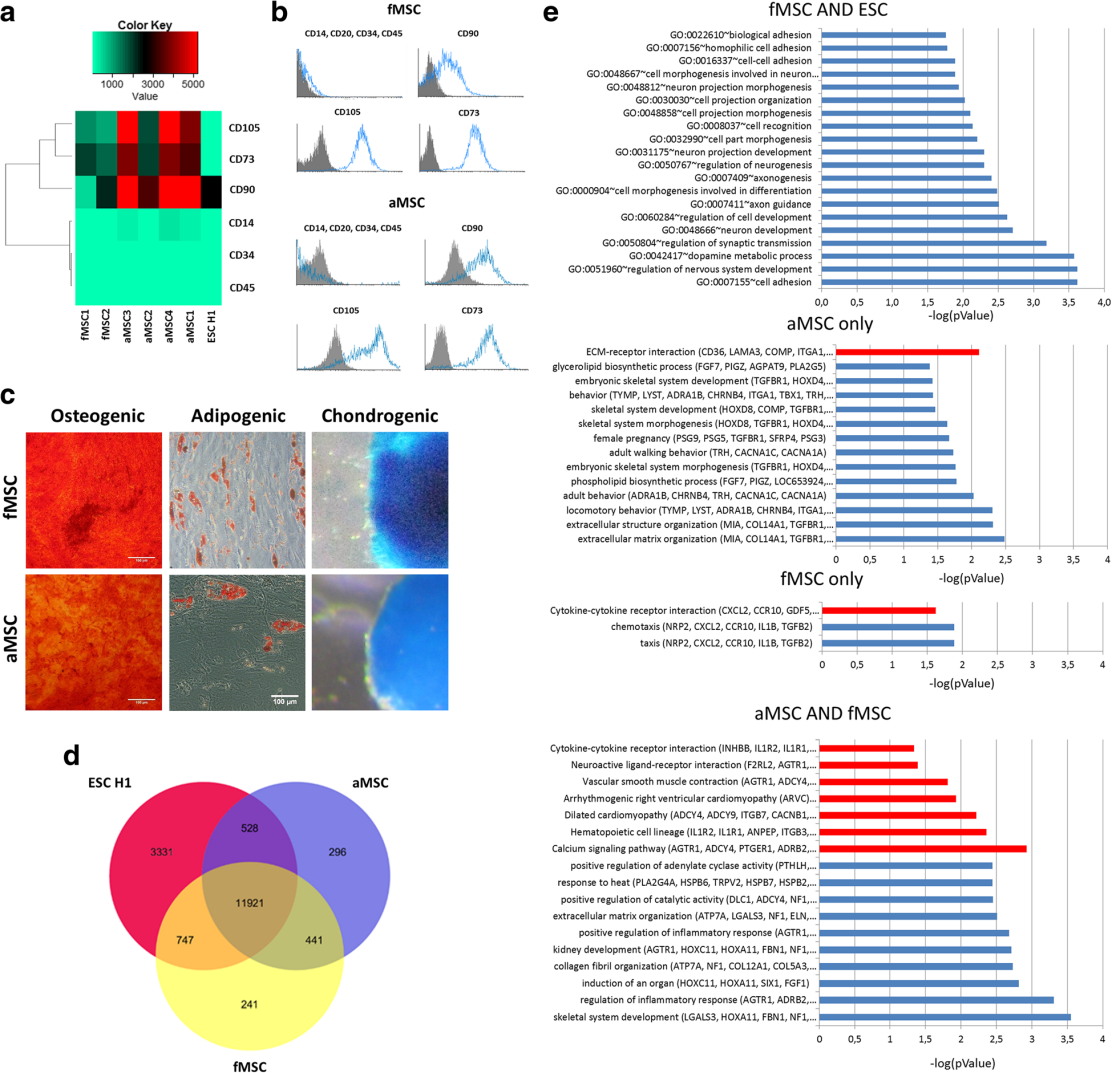
Characterization of fMSCs and aMSCs. **a** fMSCs and aMSCs express typical MSC surface marker genes CD73, CD90, and CD105 but no hematopoietic marker genes. **b** fMSCs and aMSCs show MSC-typical surface marker expression detected by FACS. Blue: fluorophore-conjugated antibody against surface antigen. Gray: isotype control. **c** Confirmation of typical MSC lineage differentiation potential in fMSCs and aMSC. Alizarin Red staining visualized the calcified matrix in red. Adipogenic: Oil Red O staining was used to visualize fat vacuoles of adipocytes in red. Chondrogenic: Cells of pellet culture were stained with Alcian blue to visualize acidic mucosubstances in blue. Pictures were taken using a stereo microscope. **d** Overlapping and distinct gene expression between fMSCs, aMSCs, and ESCs with **e** related KEGG pathways and GO terms depicted as bar diagrams with –log(pValue). KEGG pathways are marked in red and GO terms in blue

Irrespective of donor age, fMSCs and aMSCs showed a typical MSC surface marker profile by expression of CD73, CD90, and CD105 and the absence of the hematopoietic markers CD14, CD20, CD34, and CD45 at the gene expression and protein level (Fig. 1a, b). ESCs H1 which were used as a negative control did not show expression of CD73 and CD105 at the gene expression level and had a lower expression of CD90 than the MSCs. Additionally, MSCs of both age groups could be differentiated into osteoblasts, adipocytes, and chondrocytes and stained positive for Alizarin Red S (bone), Oil Red O (fat), and Alcian Blue (cartilage), respectively (Fig. 1c).

Venn diagram-based analysis of the transcriptome data revealed a higher number of genes expressed in common between fMSCs and ESCs (747 genes) compared to the overlap of aMSCs and ESCs (Fig. 1d). The 747 genes were annotated to GO terms such as cell adhesion with a *p* value below 0.01. In addition, genes expressed in common between fMSCs and aMSCs (441) were annotated to KEGG pathways such as calcium signaling and GO terms such as skeletal system development with *p* values below 0.01. Genes exclusively expressed in fMSCs (241) were annotated to the KEGG pathway cytokine-cytokine receptor interaction with a *p* value below 0.05, whereas the genes exclusively expressed in aMSCs (296) were annotated to ECM-receptor interaction and extracellular matrix organization with *p* values below 0.01 (Fig. 1e).

### Derivation and characterization of iPSCs from fMSCs and aMSCs

We previously established two iPSC lines from fetal MSCs [26], named fMSC-iPSC1 and fMSC-iPSC2. Additionally, we have described an iPSC line from MSCs of a 74-year-old donor (aMSC-iPSC1) [22]. In the present study, MSCs isolated from a 62-year-old donor were successfully reprogrammed into iPSCs (aMSC-iPSC2) as well as a new iPSC line from fMSCs was created (fMSC-iPSC3).

**Fig. 2:**
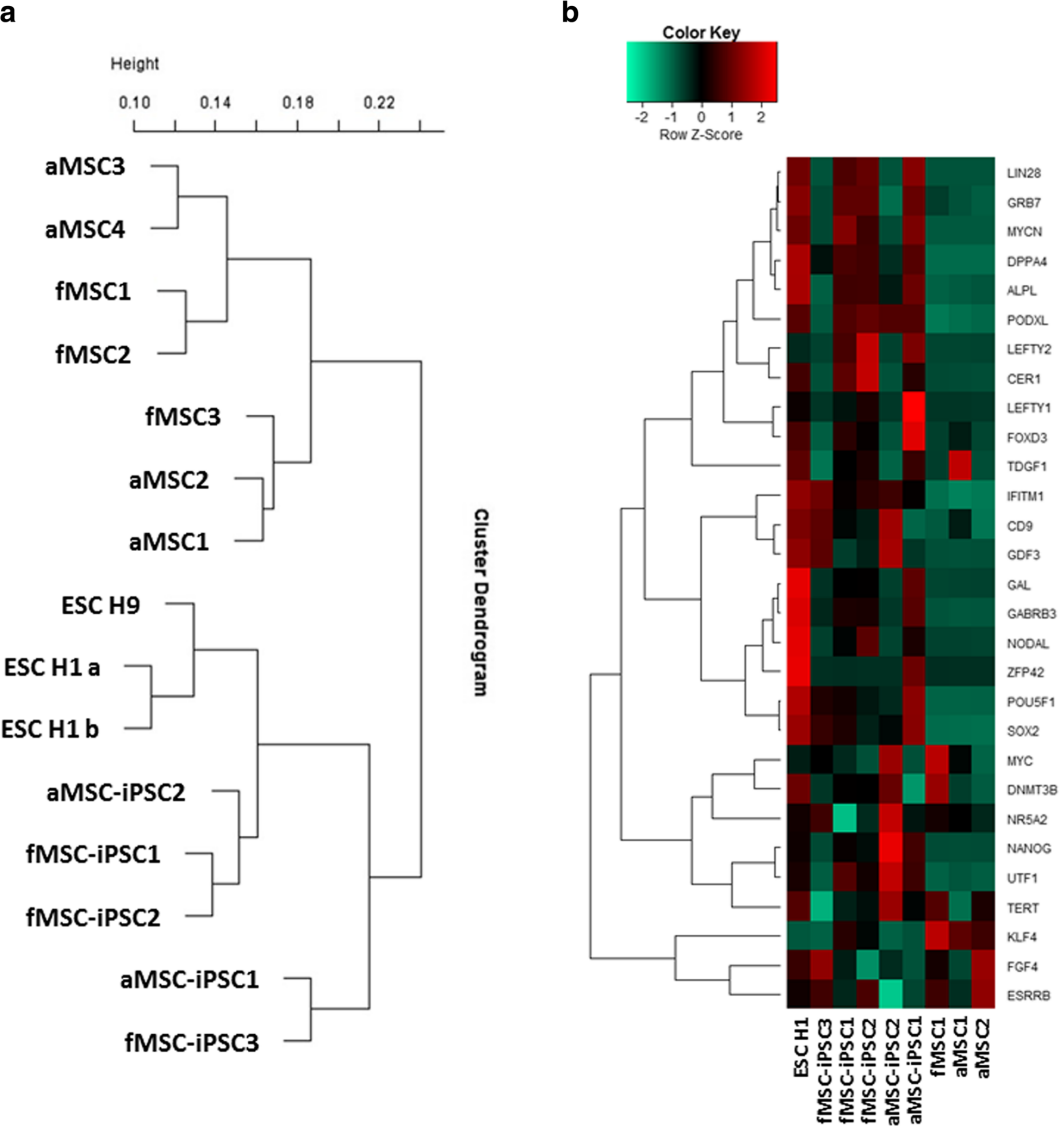
Characterization of MSC-derived iPSCs **a** Clustering analysis of primary MSCs and corresponding MSC-iPSCs and ESCs. As similarity measure Pearson correlation was used. **b** Expression of pluripotency-associated genes detected by microarray in iPSCs of fetal and aged background compared to primary MSCs and the ESC line H1

Transcriptome analysis of the native MSCs, the corresponding iPSCs, and the ESC line H1 revealed two separated clusters. The first cluster included all MSC population irrespective of the donor age which was separated from the second cluster which includes the ESCs and all MSC-iPSCs (Fig. 2a). At the transcriptome level, there was a distinct level of heterogeneity in the results since MSCs and MSC-iPSCs did not show a separation by donor-cell age. All iPSC lines expressed pluripotency-associated markers (Additional file 1: Figure S1) and a transcriptome similar to ESCs (Fig. 2b). Moreover, both fMSC-iPSC3 and aMSC-iPSC2 expressed pluripotency marker at the protein level and formed embryoid bodies and differentiated into cell types representative of the three embryonic germ layers (Additional file 1: Figure S1).

### iMSC from MSC-iPSCs of distinct age backgrounds and ESCs have MSC-typical marker expression and differentiation potential

**Fig. 3:**
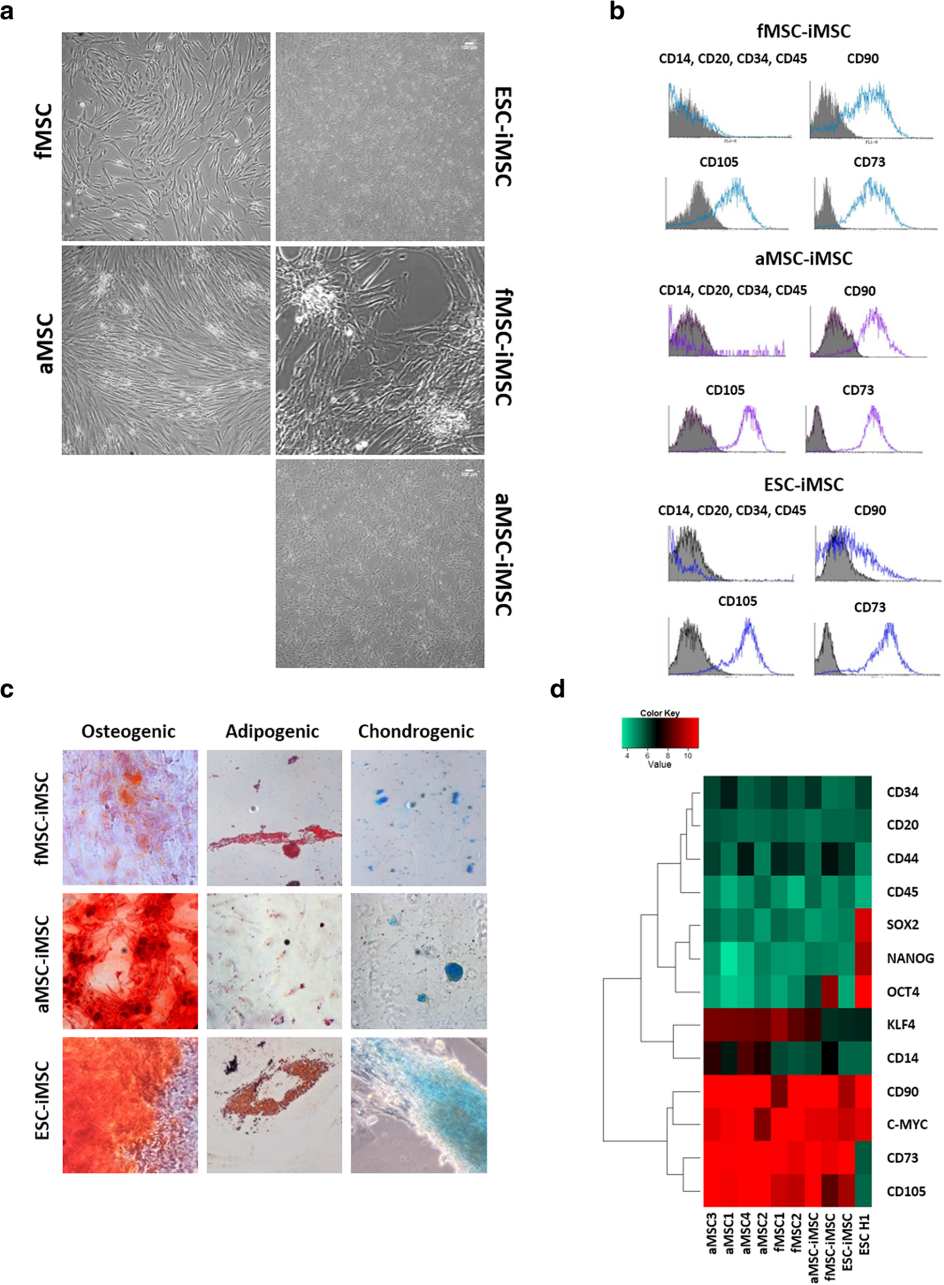
Characterization of fMSC-iMSCs, aMSC- iMSCs, and ESC-iMSCs. **a** Morphology of iMSCs compared to native MSCs. **b** Flow cytometry-based analysis of MSC surface marker in iMSCs. Blue/Purple: iMSCs labeled with antibody specific to marker. Gray: isotype control. **c** Differentiation potential of iMSCs in vitro. Osteogenic: Alizarin Red S staining; adipogenic: Oil red O staining; chondrogenic: Alcian Blue staining. **d** Gene expression of MSC, hematopoietic, and pluripotency markers in iMSCs compared to primary MSCs and ESC H1. Representative images of *n* = 3 experiments

fMSC-iPSC3, aMSC-iPSC2, and ESC line H1 were differentiated into MSCs and named fMSC-iMSCs, aMSC-iMSCs, and ESC-iMSCs. iMSCs displayed spindle-shaped morphologies comparable to primary MSCs (Fig. 3a). In addition, all iMSCs derived from primary MSCs expressed the MSC markers CD73, CD90, and CD105 but not the hematopoietic markers CD14, CD20, CD34, and CD45 (Fig. 3b). Oil Red O-positive fat droplets were detected in all iMSC preparations upon adipogenic induction. In addition, Alizarin Red-positive calcified matrix and Alcian Blue staining were detected following culture in osteogenic and chondrogenic medium, respectively (Fig. 3c). Although iMSCs were derived from pluripotent cells, they had a lower expression of pluripotency markers than the iPSC and ESCs they were derived from (Fig. 3d). Finally, comparison of the transcriptomes revealed a higher correlation co-efficient (R^2^) between iMSCs and primary MSCs (0.917–0.964) than between iMSCs and iPSCs/ESCs (0.879–0.914). Moreover, we detected a higher similarity between the transcriptomes of fMSCs and ESCs (0.925–0.939) than between aMSCs and ESCs (0.855–0.885) (Additional file 1: Figure S2).

### Primary MSCs and iMSCs have overlapping and distinct gene expression patterns revealing higher similarity between iMSCs and fMSCs

**Fig. 4:**
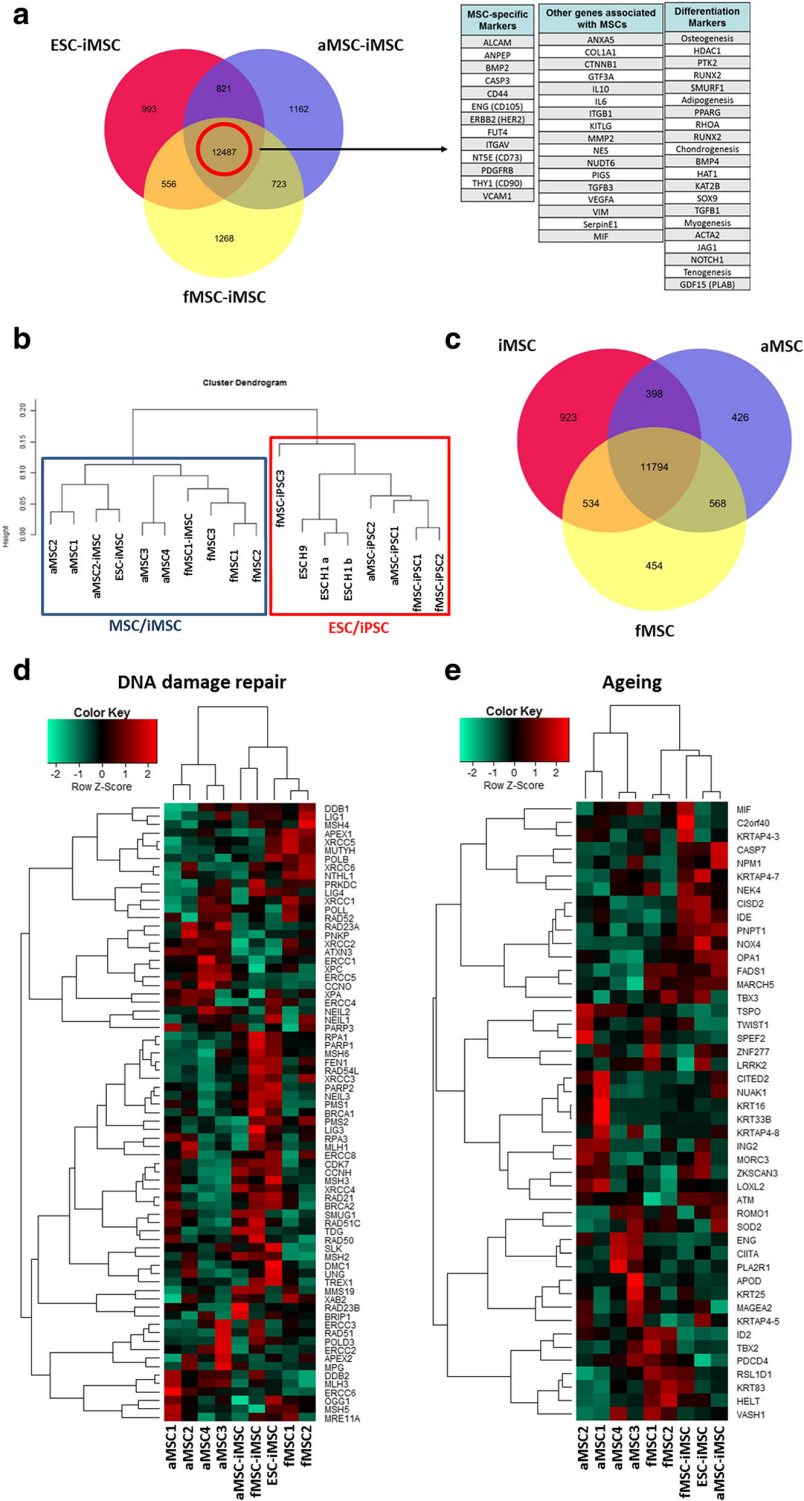
Distinct and overlapping gene expression patterns between iMSCs and primary MSCs isolated from donors of distinct ages. **a** Venn diagram-based on expressed genes detected by microarrays of one sample each of fMSC-iMSCs, aMSC-iMSCs, and ESC-iMSCs. MSC-related genes were expressed in all three iMSC preparations. **b** Clustering dendrogram of Illumina gene expression experiments based on Pearson correlation. One cluster consists of iPSCs and ESCs (red box), and the other separated cluster contains primary MSCs as well as all three iMSC preparations (blue box). **c** Venn diagram of iMSCs and MSCs from fetal and aged donors based on expressed genes of one sample each **d** Heatmap showing clustering of primary MSCs and iMSCs for genes related to DNA damage repair. **e** Heatmap showing clustering of primary MSCs and iMSCs for aging-related genes

A Venn diagram-based representation of transcriptome data identified 12,487 genes commonly expressed between the fMSC-iMSCs, aMSC-iMSCs, and ESC-iMSCs. Within this shared gene set, numerous MSC-specific genes (CD73, CD90, CD105, and PDGFRβ), MSC-associated genes (VEGFA, Vimentin, SerpinE1, and MIF), and differentiation markers (RUNX2 and PPARγ) were present (Fig. 4a). Clustering analysis of the transcriptomes resulted in the formation of two similarity-based clusters separating iMSCs (irrespective of their source) together with primary MSCs from their corresponding iPSCs and ESC samples (Fig. 4b). Another Venn diagram-based analysis comparing iMSCs (combination of fMSC-iMSCs, aMSC-iMSCs, and ESC-iMSCs), fMSCs, and aMSCs revealed that more genes were expressed in common between iMSCs and fMSCs (534 genes) than between iMSCs and aMSCs (398 genes) with the majority of genes expressed in all three groups (11794). iMSCs proved the most distinct sample set with 923 exclusively expressed genes (Fig. 4c).

Importantly, a heatmap-based clustering analysis of expression of DNA damage repair (such as FEN1 and MSH6) and aging-associated genes (such as *FADS1* and *NOX4*) revealed that iMSCs irrespective of donor age or cell type of origin are more similar to fMSCs compared to aMSCs (Fig. 4d, e).

### MSC-iMSCs acquired a rejuvenation signature

**Fig. 5:**
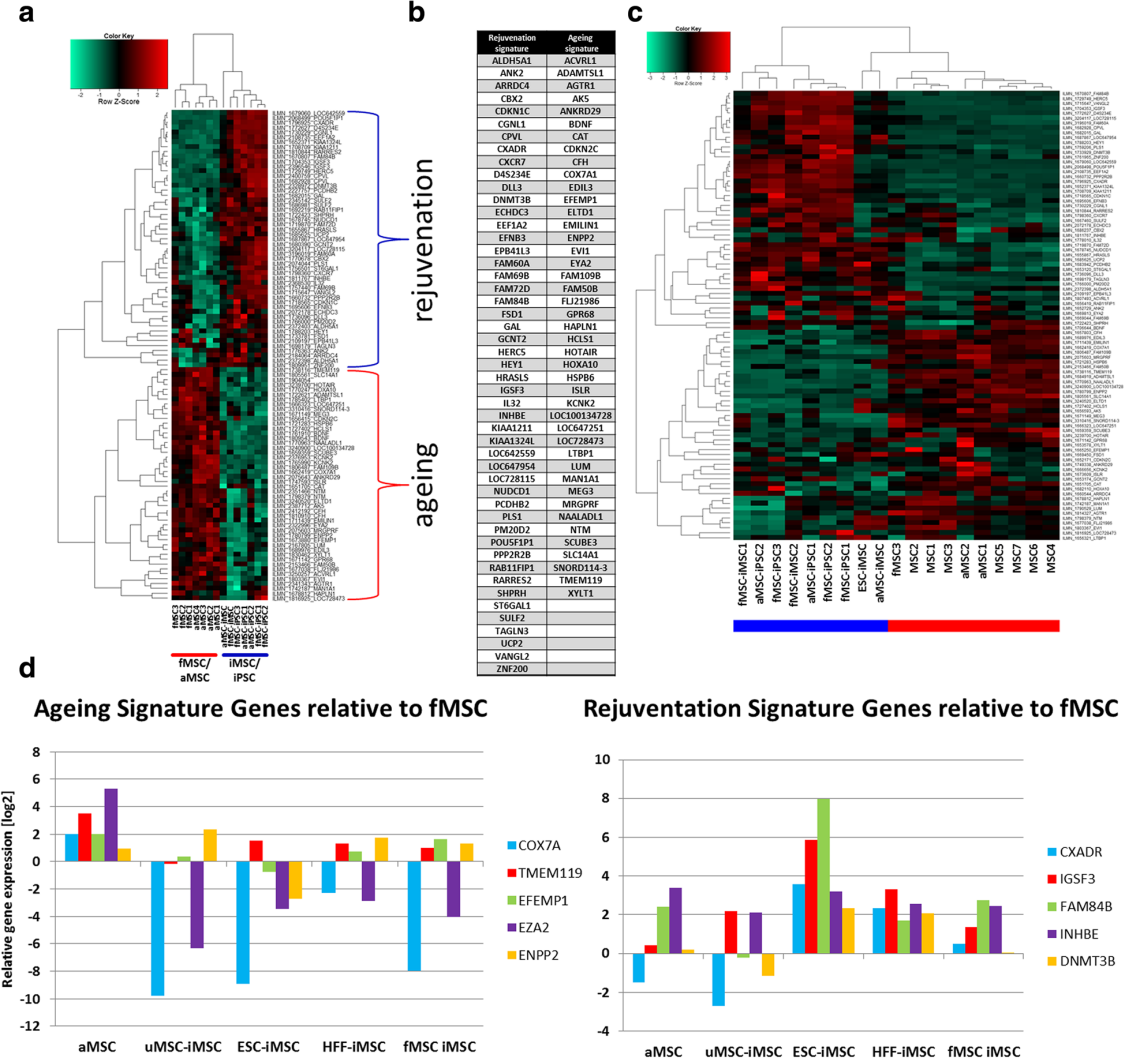
Identification of rejuvenation and aging signature. **a** Heatmap of genes expressed in MSCs but not in MSC-iMSCs and iPSCs (the aging signature) and of genes expressed in MSC-iMSCs and iPSCs but not in MSCs (the rejuvenation signature). **b** List of genes of the rejuvenation and aging signature. **c** Heatmap of genes in the rejuvenation and aging signature including published datasets [24, 25]. **d** Real-time PCR confirmation of a subset from rejuvenation and aging signature genes employing external MSC and additional iMSCs samples. Relative mRNA expression is shown normalized to expression in fMSCs

Genes expressed in iMSCs and pluripotent stem cells but not expressed in primary MSCs (fMSCs and aMSCs) were identified which we refer to as the rejuvenation signature. On a similar note, genes expressed in primary MSCs but not in pluripotent stem cells and iMSCs are referred to as the aging signature (Fig. 5a). Figure 5b shows a table based on the heatmap from Fig. 5a with the gene names within the rejuvenation and aging signature. To validate our rejuvenation and aging signatures, we carried out an additional analysis incorporating already published datasets of primary human MSCs of different ages [24, 25]. A hierarchical clustering analysis of gene expression including the new samples (MSC1–7) independently confirmed the validity of our rejuvenation signature (e.g., *PM20D2* and *HRASLS*) and aging signature (e.g. *FAM109B* and *EDIL3*) reflecting the respective expression levels (Fig. 5c).

For further verification of the rejuvenation signature, real-time PCR analysis was carried out using RNA from additional independent adult MSC (56 years) and iMSC samples from distinct age groups (urine-derived iPSC-derived iMSCs (51 years); ESC-iMSCs (prenatal), fMSC-iMSCs (prenatal), and HFF-iMSCs (human fetal foreskin-derived iPSC-derived iMSCs)) employing primers for genes of the rejuvenation (*IGSF3*, *CXADR*, *FAM84B*, *INHBE*, and *DNMT3B)* and aging signature (*COX7A*, *EZA2*, *EFEMP1*, *ENPP2*, and *TMEM119*) (Fig. 5d). For *IGSF3*, the mRNA expression level in all iMSC preparation was higher than that in the fMSCs and aMSC samples whereas only three of the four iMSC samples showed increased *CXADR* expression levels. For *DNMT3B*, a rejuvenation signature, two of the four iMSC samples showed upregulation. The other two genes of the rejuvenation signature showed comparable levels in aMSCs and iMSCs. For the aging signature, *COX7A*, *EZA2*, *EFEMP1*, and *TMEM119* were expressed at lower levels in iMSCs than in aMSCs with the exception of *ENPP2* (Fig. 5d).

### Protein association network analyses confirm rejuvenation and aging signature

**Fig. 6:**
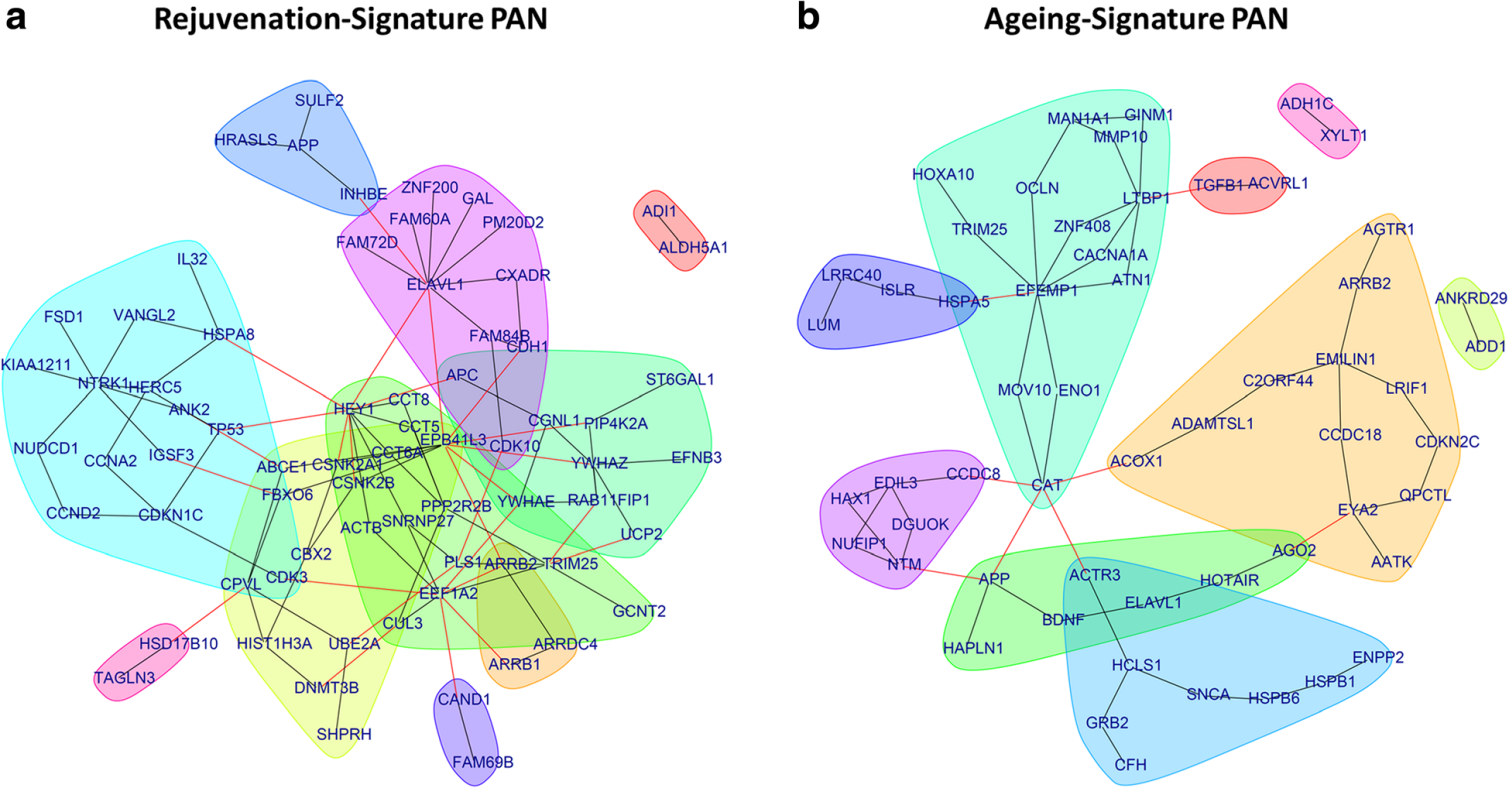
Protein association network related to aging and rejuvenation signatures of gene expression in iMSCs and parental MSCs. Based on the Biogrid database protein interaction, networks have been constructed from the rejuvenation signature (**a**) and aging signature (**b**) listed in Fig. 5b. Most of the proteins coded by genes from both signatures can be connected to a network with interactions reported in the Biogrid database. Community clustering of the network via edge-betweenness reveals several communities with densely connected nodes and fewer connections across communities. Communities are indicated by dedicated color shading; edges between communities are colored red. **a** The rejuvenation signature PAN includes communities characterized by INHBE (blue), TP53, CDKN1C, IL32 (light blue), CDK10 (petrol), ELAVL1 (purple), DNMT3B (yellow), and EEF1A2 (green). **b** The aging signature PAN includes communities characterized by HSPA5 (blue), CCDC8 (purple), CAT (petrol), EYA2 (yellow), APP (green), and TGFB1 (red)

Using the genes of the rejuvenation signature as input, a protein association network (PAN) was created adding the *n* = 30 interaction partners with the most interactions from the Biogrid database. We used community clustering to identify densely connected groups of proteins with fewer connections across groups. The rejuvenation signature PAN (Fig. 6a) includes communities characterized by INHBE (blue), TP53, CDKN1C, IL32 (light blue), CDK10 (petrol), ELAVL1 (purple), DNMT3B (yellow), and EEF1A2 (green).

Analogously, we generated a PAN based on the aging signature which revealed genes involved in the TGFβ and mTOR-signaling pathways as well as factors associated with oxidative stress including CAT (Fig. 6b). The aging signature PAN (Fig. 6b) includes communities characterized by HSPA5 (blue), GRB2 (light blue), CCDC8 (purple), CAT (petrol), EYA2 (yellow), APP (green), and TGFB1 (red).

### iMSCs of different age backgrounds show overlapping secretomes with fetal MSCs

**Fig. 7:**
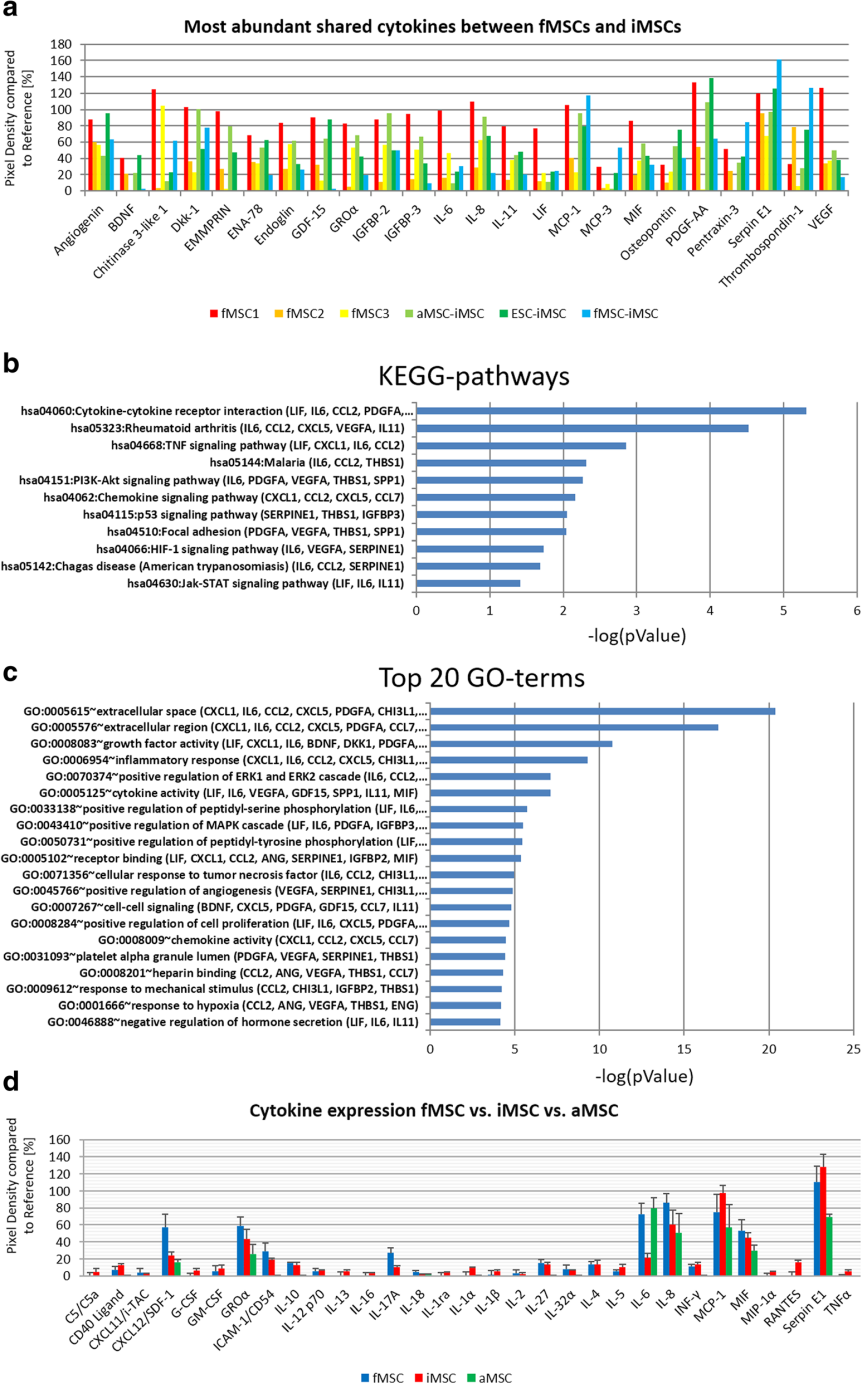
Comparative analyses of the secretome of fMSCs, iMSCs, and aMSCs. **a** Cytokine expression of fMSC1, fMSC2, fMSC3, and iMSCs (fMSC-iMSCs, aMSC-iMSCs, and ESC-iMSCs) detected using membrane-based cytokine arrays. Expression plot of the most abundant cytokines shared between fMSCs and iMSCs; threshold of expression in comparison to reference spots was set to 20%. **b** KEGG pathway analysis of common cytokines between fMSCs and iMSCs–log(pValue). **c** GO terms associated with common secreted cytokines between fMSCs and iMSCs as –log(pValue). **d** Cytokine expression of fMSCs, iMSCs, and aMSCs (aMSC5, aMSC6, aMSC7)

Based on a cytokine array, the secretomes of fMSC-iMSCs, aMSC-iMSCs, and ESC-iMSCs were found to be similar to the secretomes of primary fMSC1, fMSC2, and fMSC3 (Fig. 7a, b). iMSCs, independent from their origin, as well as fetal MSCs showed a large number overlap in the most abundantly secreted cytokines: angiogenin, BDNF, Chitinase 3-like 1, Dkk-1, EMMPRIN, ENA-78, endoglin, GDF-15, GROα, IGFBP-2, IGFBP-3, IL-6, IL-8, IL-11, LIF, MCP-1, MCP-3, MIF, osteopontin, PDGF-AA, pentraxin-3, serpin E1, thrombospondin-1, and VEGF (Fig. 7a, Additional file 1: Figure S3). KEGG pathway analysis of the common secreted cytokines showed their involvement in processes like cytokine-cytokine receptor interaction, TNF signaling pathway, chemokine signaling pathway, PI3K-Akt signaling pathway, HIF-1 signaling pathway, and Jak-STAT signaling pathway (Fig. 7b). Gene Ontology analysis of the common secreted cytokines showed involvement in processes such as regulation of growth factor activity, inflammatory response, positive regulation of ERK1 and ERK2 cascade, positive regulation of angiogenesis, and cell proliferation (Fig. 7c). In addition to this, the secretomes of fMSCs and iMSCs were compared to that of aMSCs (Fig. 7d, Additional file 1: Figure S3). In comparison to fMSCs and iMSCs (independent of the source), MSCs from aged individuals (aMSCs) secreted fewer cytokines and at lower levels except for IL6.

## Discussion

Derivation and characterization of pluripotent stem cell-derived MSCs (iMSCs) are on the rise [18, 44–46]. iMSCs have been shown to enhance regeneration and healing when applied to a variety of animal models; multiple sclerosis, limb ischemia, arthritis, liver damage, bone defects, wound healing, and hypoxic-ischemia in the brain [46–53]. In this study, we comparatively and critically assessed the effect of donor age and cell type specificity on the iMSC “rejuvenated” signature based on transcriptome analysis and further studied their paracrine signaling potential by secretome analyses. We revealed that fMSCs share a higher transcriptome similarity with ESCs than with aMSCs. This age-related difference may be due to genes involved in cell adhesion (Fig. 1e), which is in agreement with the reported role of adhesion-related processes in pluripotent stem cells [54]. However, the iMSCs generated in this study met the criteria defined for primary MSCs to a certain extent in terms of morphology and surface marker expression (Fig. 3a,b), as previously shown for iMSC generation from fibroblast-derived iPSCs [18]. In agreement with the MSC criteria [2], the generated iMSCs were able to differentiate into bone, cartilage, and fat cells in vitro. In addition, we could successfully confirm a high level of similarity between primary MSCs and iMSCs on transcriptome level and could show that these iMSCs although originating from pluripotent cells are not pluripotent themselves (low similarity to iPSCs) which is important for potential use in future clinical applications.

The expression patterns of genes associated with aging and DNA damage repair in all iMSC populations clustered closer to fMSCs than to aMSCs (Fig. 4d, e), thus indicating a rejuvenation. DNA damage has been shown to be associated with the complex process of aging before [55]. Irrespective of donor age and cell source, iMSCs acquired a rejuvenation gene signature also present in pluripotent stem cells but not in the parental MSCs (Fig. 5a, b). Conversely, we observed a gene set representing the aging signature comprising genes expressed in primary MSCs but not in pluripotent stem cells and iMSCs. We could independently confirm the extracted aging and rejuvenation signature by including already published datasets of adult MSCs [24, 25] in similarity analyses based on both gene sets (Fig. 5c). Further confirmation of the signatures was carried out at the mRNA level using additional MSC and iMSC samples (Fig. 5d). A large number of the genes within the rejuvenation signature play important roles in embryonic tissues and in development thus indicating the presence of features associated with early development in iMSCs, and therefore, it would appear, endowing iMSCs with enhanced regenerative properties. The rejuvenation signature PAN revealed communities characterized by INHBE, TP53, CDKN1C, IL32, CDK10, ELAVL1, DNMT3B, and EEF1A2. INHBE participates in the activin/nodal branch of the TGFB signaling pathway which is needed for maintenance of pluripotency [56, 57]. CDK10, CDKN1C, and TP53 are involved in cell cycle control which obviously plays an important role in stem cell self-renewal [58]. However, the detailed cell cycle coordination in order to determine cell fates is not fully uncovered. CDK10 like all members of the CDK family is responsible for cell cycle progression but is limited to the G2-M phase which Vallier et al. describe as necessary to block pluripotency upon induction of differentiation referring to Gonzalez et al. [59]. Gonzales et al. furthermore report that the ATM/ATR-CHEK2-TP53 axis enhances the TGFβ pathway to prevent pluripotent state dissolution. In a previous publication, we reported compromised induction of pluripotency in fibroblasts from a Nijmegen Breakage syndrome patient under conditions of impaired DNA damage repair and downregulated TP53 and cell cycle genes [60]. CDKN1C reduces cell proliferation by inhibiting cyclin/CdK complexes in the G1 phase [61] and is a major regulator of embryonic growth as has been reported by Andrews et al. for the imprinted domain on mouse distal chromosome 7 [62]. ELAVL1 (HuR) has been associated with regulation of growth and proliferation of vascular smooth muscle cells [63]. DNMT3B has been reported to be essential for de novo methylation and mammalian development [64] and DMNT1 and DNMT3B were shown to decrease upon aging [65].

The aging signature PAN includes communities characterized by HSPA5, CCDC8, CAT, EYA2, APP, and TGFB1. Catalase (CAT) is an antioxidant which has been reported to have decreased activity upon aging in rats [66]. GRB2 is part of the mTOR-signaling pathway which coordinates eukaryotic cell growth and metabolism with environmental inputs [67]. TGFβ-signaling plays a major role in young and aging organisms but changes its functionality. Baugé et al. describe a shift of TGFβ-signaling from SMAD2/3 to SMAD1/5/8 as cause of a shift from chondrogenic differentiation and maturation in young joints to hypertrophic differentiation in aged or osteoarthritic joints [68]. BDNF has been reported to regulate the amyloid precursor protein APP [69] that is involved in activity-dependent synaptic plasticity and is upregulated after birth but then stays unchanged during aging in rat hippocampus [70].

Accordingly, the loss of the aging signature during iMSC derivation likely contributed to the advantageous features of iMSCs compared to primary MSCs. The observed fetal-like expression pattern of genes involved in DNA damage repair in iMSCs could be due to the involvement of this process in pluripotency induction. An alternative explanation could be that young or progenitor cells have a better capacity to repair DNA damage [71].

A rejuvenated state of processes involved in aging in iMSCs is furthermore likely as the epigenetic rejuvenation of MSCs through pluripotency induction has been described [13].

Of considerable potential significance, from a regenerative medicine perspective, iMSCs should have a similar secretome to that of the corresponding parental MSCs. In line with this, the secretion of GROα, IL-6, IL-8, MCP-1, MIF, SDF-1, and Serpin E1 in bone marrow-derived MSCs has been described [72]. A further study showed that MSCs derived from bone marrow secrete angiogenin, G-CSF, GM-CSF, GROα, IL-1α, IL-6, IL-8, INFγ, MCP-1, oncostatin M, RANTES, and TGFβ and do not secrete IL-2, IL-4, IL-10, IL-12, IL-13, MIP-1β, and SDF-1α [73], all in agreement with our iMSC secretome profile. Interestingly, we detected the secretion of anti-inflammatory and pro-inflammatory cytokines in iMSCs and fetal mesenchymal populations confirming findings in MSCs [74]. Gene Ontology analysis revealed overlapping capabilities to interact with the immune system and involvement in regeneration processes of fetal MSCs and iMSCs corroborating studies with MSCs derived from adult donors [75–77]. A KEGG pathway analysis revealed involvement of the overlapping secreted cytokines between fetal MSCs and iMSCs in TNF signaling pathway, Jak-STAT signaling pathway, and PI3K-Akt signaling pathway. Interestingly, we found SerpinE1, thrombospondin-1, IGFBP3, endoglin, and angiogenin to be abundantly secreted in fetal MSCs and iMSCs. The described role of SerpinE1, thrombospondin-1, and endoglin in wound healing [78–80] indicate a putative fetal-like feature of wound healing properties of iMSCs in vivo [81, 82]. Our analyses revealed that aMSCs compared to fMSCs and iMSC secrete a reduced repertoire of cytokines and at significantly lower levels. However, fMSCs, aMSCs, and iMSCs secrete comparable levels of IL6, IL8, SDF-1, MCP-1, MIF, Serpin E1, and GROα. This once again reinforces the notion that MSCs isolated from the elderly may not be as potent as fetal MSCs and pluripotent stem cell-derived MSCs.

## Conclusions

In summary, the current study shows that MSCs of fetal and aged background are not identical and MSCs generated from iPSCs (iMSCs) bear typical characteristics of native MSCs but more in common with fetal MSCs. The key finding from our study is the identification of a rejuvenation gene signature in iMSCs (irrespective of donor age) which also is present in pluripotent stem cells but not in the parental MSCs. Most important for regenerative medicine, iMSCs irrespective of initial age re-acquire a more similar secretome to that of fetal MSCs than aged MSCs. In conclusion, our findings show that the acquisition of a rejuvenated phenotype in iMSCs re-enforces the utility of the “iMSC concept” in regenerative medicine and cell replacement therapy in an ever increasing aging population.

## Additional file

Additional file 1: **Table S1.** List of primary MSC samples. Table S2. List of primers. **Table S3.** List of antibodies. Figure S1 Pluripotency marker staining of generated iPSC line from fMSCs and aMSCs as well as EB formation. **Figure S2.** Correlation coeficiency table. Figure S3 Cytokine membranes. (DOCX 2493 kb)

## Abbreviations

aMSC: Adult MSC
CD: Cluster of differentiation
DAVID: Database for Annotation, Visualization and Integrated Discovery
EB: Embryoid body
ESCs: Embryonic stem cells
FACS: Fluorescence-activated cell sorting
fMSC: Fetal MSC
GEO: Gene Expression Omnibus
GO: Gene Ontology
iMSCs: iPSC-derived mesenchymal stromal cells
iPSCs: Induced pluripotent stem cells
KEGG: Kyoto Encyclopedia of Genes and Genomes
MSCs: Mesenchymal stem cells
PAN: Protein association network
