# Supplementary material for: Human iPSC-derived MSCs (iMSCs) from aged individuals acquire a rejuvenation signature
Source: Stem Cell Res Ther. 2019 Mar 18;10:100. doi: 10.1186/s13287-019-1209-x (PMC6423778; doi:10.1186/s13287-019-1209-x)
Supplement: Supplementary file 1 — Table S1. List of primary MSC samples. Table S2. List of primers. Table S3. List of antibodies. Figure S1 Pluripotency marker staining of generated iPSC line from fMSCs and aMSCs as well as EB formation. Figure S2. Correlation coeficiency table. Figure S3 Cytokine membranes. (DOCX 2493 kb) [file 13287_2019_1209_MOESM1_ESM.docx]

**Human iPSC-derived MSCs (iMSCs) from aged individuals acquire a rejuvenation signature**

Lucas-Sebastian Spitzhorn^1*^, Matthias Megges^1*^, Wasco Wruck^1^, Md Shaifur Rahman^1^, Jörg Otte^1^, **Özer Degistirici^2^,** Roland Meisel^2^, Rüdiger Volker Sorg^3^, Richard O.C. Oreffo^4^, and James Adjaye^1#^

^1^Institute for Stem Cell Research and Regenerative Medicine, Medical Faculty, Heinrich Heine University, Düsseldorf, Moorenstr. 5, 40225 Düsseldorf, Germany

^2^Division of Paediatric Stem Cell Therapy, Clinic for Pediatric Oncology, Hematology and Clinical Immunology, Medical Faculty, Heinrich Heine University, Düsseldorf, Moorenstr. 5, 40225 Düsseldorf, Germany

^3^Institute for Transplantation Diagnostics and Cell Therapeutics, Heinrich Heine University Hospital, Moorenstr. 5, 40225, Düsseldorf, Germany

^4^Bone and Joint Research Group, Centre for Human Development, Stem Cells and Regeneration, Institute of Developmental Sciences, University of Southampton, Southampton SO16 6YD, UK

^*^ Matthias Megges and Lucas-Sebastian Spitzhorn contributed equally to this work.

Lucas-Sebastian Spitzhorn: lucas-sebastian.spitzhorn@hhu.de

Matthias Megges: matthiasmegges@gmail.com

Md Shaifur Rahman: shaifur.rahman@med.uni-duesseldorf.de

Wasco Wruck: wasco.wruck@med.uni-duesseldorf.de

Richard O.C. Oreffo: roco@soton.ac.uk

James Adjaye: james.adjaye@med.uni-duesseldorf.de

Jörg Otte: Joerg.Otte@med.uni-duesseldorf.de

Roland Meisel: meisel@med.uni-duesseldorf.de

Özer **Degistirici: Degistirici@med.uni-duesseldorf.de**

**Rüdiger Volker Sorg: Ruediger.Sorg@med.uni-duesseldorf.de**

**^#^ Corresponding author**

Prof. Dr. James Adjaye

Email: James.Adjaye@med.uni-duesseldorf.de

**Supplementary Data**


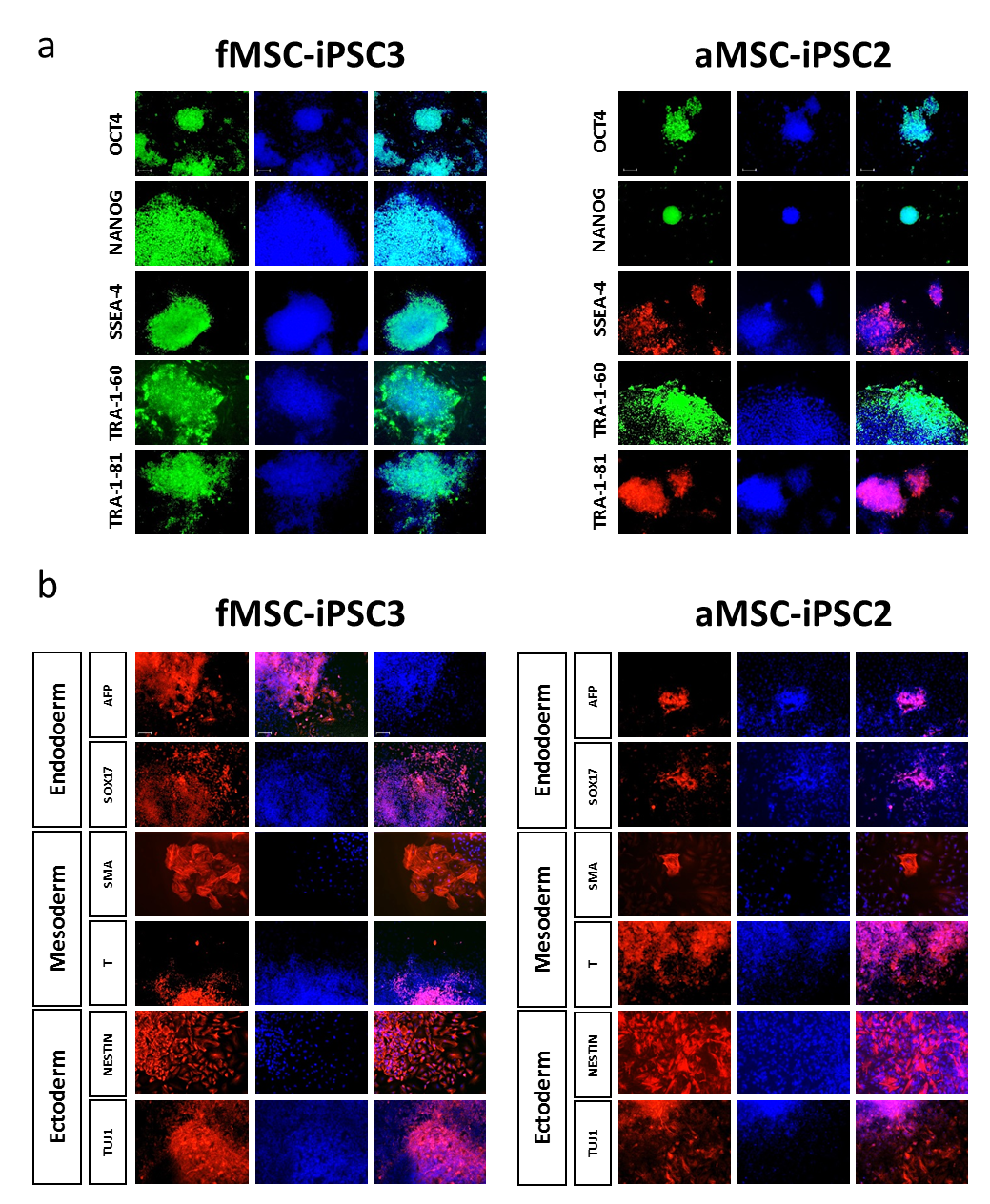


**Fig. S1:** **Pluripotency marker staining of generated iPSC line from fMSCs and aMSCs as well as EB formation.** (a) Pluripotency marker expression of newly established fMSC-iPSC3 and aMSC-iPSC2. Cell nuclei are stained with DAPI. (b) Embryoid body formation and staining of three germ layer structures of the newly established iPSC lines.


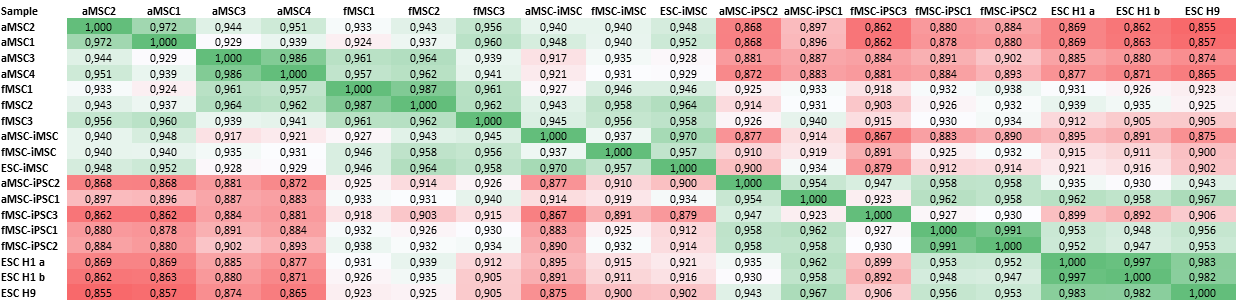


**Fig. S2: Correlation Coeficiency table.** Pearson Correlation coefficients of native MSCs, iPSCs, ESCs and iMSCs.


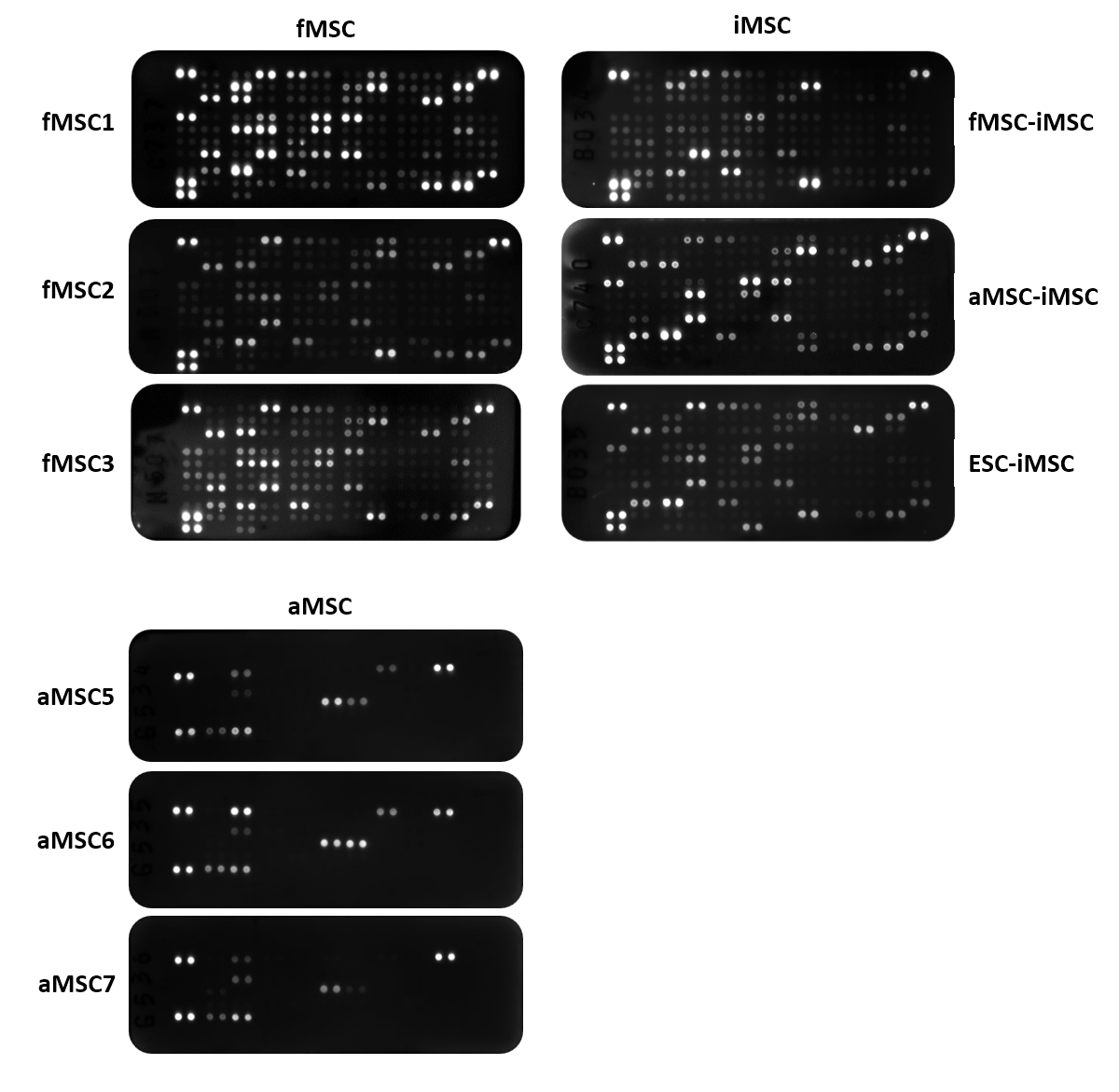


**Fig. S3: Cytokine membranes.** Cytokine membranes of the secretome analysis of fMSCs, iMSCs and aMSCs.

**Table S1: List of primary MSC samples**

| **Sample** | **Name** |
| --- | --- |
| Fetal femur 55 days post conception | fMSC1 |
| Fetal femur 55 days post conception | fMSC2 |
| Fetal femur 55 days post conception | fMSC3 |
| Bone-marrow MSCs from 74 year old | aMSC1 |
| Bone-marrow MSCs from 62 year old | aMSC2 |
| Bone-marrow MSCs from 60 year old | aMSC3 |
| Bone-marrow MSCs from 70 year old | aMSC4 |
| Bone-marrow MSCs from 62 year old | aMSC5 |
| Bone-marrow MSCs from 64 year old | aMSC6 |
| Bone-marrow MSCs from 69 year old | aMSC7 |
| Previously published adult MSC [24] | MSC1 |
| Previously published adult MSC [24] | MSC2 |
| Previously published adult MSC [24] | MSC3 |
| Previously published adult MSC (29 years) [25] | MSC4 |
| Previously published adult MSC (48 years) [25] | MSC5 |
| Previously published adult MSC (60 years) [25] | MSC6 |
| Previously published adult MSC (76 years) [25] | MSC7 |

**Table S2: List of primers**

| **Gene** | **forward primer sequence (5´-3´)** | **reverse primer sequence (5´-3´)** |
| --- | --- | --- |
| CXADR | AACGTTGTCCCTCCTTCAAA | ATGAGCGCTAGAGCAAGCAA |
| IGSF3 | GAGTGGCAGATTGTTGGGGA | GCGAAGCCCATTTCAAGAGC |
| FAM84B | CTCTCCGCGGGTAGCCT | CGAAACCAACTCCAGGGTCA |
| INHBE | TCTAGTGGCTTGAGGGGTGA | GCTGTTGCCTTCTAGGGGTC |
| DNMT3B | GCTCACAGGGCCCGATACTT | GCAGTCCTGCAGCTCGAGTTTA |
| COX7A | ATCCCGTTGTACCTGAAGGG | TGTAGACAGTGCCGCCCA |
| TMEM119 | CAAGGAACTGGTCCTGGGG | CAGGAGCAGCAACAGAAGGA |
| EFEMP1 | GCCGCACAGGTATTTTTGCT | TGTCCTGTGACTTGACCAGC |
| ENPP2 | CCAACCATGCCAGAGGAAGT | CCAACTTGTTCTTTGGCTCTACC |
| EYA2 | GGAGTGTGCACCAGGACTAT | GAGGGTTGTAGGATGAGCCG |
| RPL37A | GTGGTTCCTGCATGAAGACAGTG | TTCTGATGGCGGACTTTACCG |

**Table S3: List of antibodies**

| **Primary Antibodies** | **Providing company** | **Dilution** |
| --- | --- | --- |
| OCT4 | Santa Cruz ( #sc-5279) | 1:100 |
| SOX2 | Santa Cruz (#sc-17320) | 1:100 |
| KLF-4 | Santa Cruz ( #sc-20691) | 1:100 |
| SSEA4, TRA-1-60 and TRA-1-81 | Merck Millipore (#SCR004) | 1:100 |
| NANOG | Abcam (#ab62734) | 1:100 |
| Alpha-Fetoprotein, (AFP) | Sigma-Aldrich (#WH0000174M) | 1:100 |
| SOX17 | R&D (#AF1924) | 1:50 |
| Nestin | (Chemicon (#MAB5326) | 1:200 |
| β-Tubulin III | Sigma-Aldrich (#T8660) | 1:1000 |
| Smooth-Muscle-Actin (SMA) | Dako (#M0851) | 1:100 |
| Brachyury (T) | R&D (#AF2085) | 1:50 |
| Mouse Alexa-488 | Life Technologies (#A11001) | 1:300 |
| against rabbit IgG | Life Technologies (#A11015) | 1:300 |
| goat IgG | Life Technologies (#A11006) | 1:300 |
| Alexa-594 against mouse IgG | Life Technologies (# A11032) | 1:300 |
| against rabbit IgG | Life Technologies (#A11012). | 1:300 |
